# Supplementary material for: A novel single-cell based method for breast cancer prognosis
Source: PLoS Comput Biol. 2020 Aug 24;16(8):e1008133. doi: 10.1371/journal.pcbi.1008133 (PMC7470419; doi:10.1371/journal.pcbi.1008133)
Supplement: S1 File — (PDF) [file pcbi.1008133.s001.pdf]

Supplementary information of "A novel single-cell based method for breast cancer prognosis"

Xiaomei Li[1], Lin Liu[1], Gregory J. Goodall[2,3], Andreas Schreiber[2], Taosheng Xu[4], Jiuyong Li[1] and Thuc D. Le[1,*]

1 UniSA STEM, University of South Australia, Mawson Lakes, SA, Australia
2 Centre for Cancer Biology, an alliance of SA Pathology and University of South Australia, Adelaide, SA, Australia
3 School of Medicine, Discipline of Medicine, University of Adelaide, SA, Australia
4 Institute of Intelligent Machines, Hefei Institutes of Physical Science, Chinese Academy of Sciences, Hefei, 230031, China
* Thuc.Le@unisa.edu.au

1 Benchmark methods

Table A summarizes the key information of six benchmark methods in breast cancer prognosis. *PAM50* starts with an extended intrinsic gene set from previous studies, then selects genes based on their contributions in terms of distinguishing the five intrinsic breast cancer subtypes [1]. *Mamma* infers the differentially expressed genes and ranks them based on the correlation between gene expression profiles and survival outcomes [2]. *RS* selects 16 gene signatures which are associated with the distant recurrence of patients from 250 published candidate genes [3]. *GGI97* ranks genes according to their differentially expressed between histologic grade 1 and 3 tumors [4]. *Endo* conducts uni-variable Cox regression and finally chooses 8 genes of interest [5]. *LM* analyzes transcriptomics in the parental MDA-MB-231 and the LM2 cell lines and identifies 54 unique genes associated with lung metastagenicity and virulence [6].

Table A: **The description of benchmark methods.**

Method	Platform	#transcript	#gene	Function enrichment of signatures
PAM50 [1]	Agilent array	50	50	cell cycle regulation, nuclear division and proliferation
Mamma [2]	Agilent array	70	66	cell cycle regulation, proliferation and spindle localization
RS [3]	RT-PCR	16	16	proliferation, invasion, ER and HER2
GGI97 [4]	Affymetrix array	128	97	cell cycle regulation and proliferation
Endo [5]	qRT-PCR and Affymetrix array	8	8	proliferation, apoptosis, cell adhesion, and cell signaling
LM [6]	Affymetrix array	54	54	EREG, chemokine, the matrix metalloproteinases, cell adhesion and receptor

This table shows the platform, number of signatures (transcripts, and the mapping genes) and the functional enrichment of signatures in each method. The number of genes within a method is less than the number of transcripts because some genes are duplicated with different probe names.

2 Experiment details

In the pre-processing step, we use the *magic()* function in the *RMAGIC* package to denoise and impute the value for all genes. The function requires three input parameters: the number of PCA components *npca*, the number of nearest neighbors in the adaptive Gaussian kernel *ka*, the number of times for the exponentiation of Markov affinity matrix *t*. In this study, we use the default parameters suggested in the paper [7]. Then, we extract the fully smoothed data matrix and filter out low expression genes and genes expressed in less than 20% cells. After pre-processing, the scRNA-seq data after filtering consists of 78 EMT markers and 3443 other genes.

In step 2 of *scPrognosis*, we need to infer a linear trajectory as EMT pseudotime. Similar to the paper [7], we can simply use the expression profile of *VIM* as the proxy for EMT pseudotime, which is named VIM-time to distinguish from the pseudotime inferred by a trajectory algorithm. We use the method *Wanderlust* [8] to identify EMT pseudotime (named W-time) based on pre-defined EMT markers from 315 general EMT markers in cancers [9]. Dropout events may lead to a lack of detection of expressed EMT markers, which obscures the relationship between EMT markers and dynamic EMT trajectory. Thus we estimate W-time by running *Wanderlust* on a set of EMT markers with the hyper-variance(10 genes for epithelial markers and 10 genes for mesenchymal markers). There are four key parameters in *Wanderlust*: the number of nearest neighbors *k*, the start point, the number of neighbors selected for each node in a k-nearest neighbors graph *l* and the number of l-out-of-k-nearest neighbors graphs *ng*. In this study, the start point is set to a set of cells with high expression of epithelial markers and low mesenchymal markers. The parameters *k*, *l* and *ng* are set to 60, 12 and 5, respectively.

Based on the obtained EMT pseudotime, we can construct a dynamic gene co-expression network from scRNA-seq data. Each node of the network represents a gene. we use the *MAC_perm()* function in *LEAP* [10] package to determine the cutoff θ which is used to identify if there is an edge between two nodes. *MAC_perm()* estimates the false discovery rate (FDR) by the ratio

of the average number observed in the permuted datasets to the number of observed correlations at a cutoff. We can get the θ when the FDR is less than 0.05.

3 Integrative method is better than individual methods

In this section, we investigate the performance of cancer prognosis using our integrative method (scP.V and scP.W) and three individual methods (MAD, SDE, and NET). SDE and NET rely on the pseudotime, so we use prefix notations of these methods to indicate the utilized pseudotime in the experiment. scP.V and scP.W are two versions of the proposed integrative method that uses different pseudotimes, VIM-time and W-time, respectively. For each method, we extract its top 50 ranked genes for validation. Table B summarizes the mean C-index produced by 100 runs of 10-fold cross-validation on each dataset. From the results, we observe that most of the C-indices reported here are bigger than 0.5, which means scRNA-seq based methods effectively predict the risk scores of patients in most cases. According to the mean ranks, the order of methods is $scP.W \succ scP.V \succ W.SDE \succ VIM.SDE \succ VIM.NET \succ W.NET \succ MAD$. That means the integrative method outperforms individual methods.

Table B: **Performance comparison of cancer prognosis using integrative method and individual methods based on scRNA-seq data.**

	MAD	VIM.SDE	VIM.NET	W.SDE	W.NET	scP.V	scP.W
TCGA(OS)	0.53	0.52	0.56	0.59	0.59	**0.62**	0.60
TCGA(RF)	0.48	0.53	0.54	0.65	0.53	0.56	**0.66**
METABRIC(OS)	0.58	0.57	0.58	0.56	0.57	0.59	**0.59**
METABRIC(RF)	0.58	0.61	0.60	0.61	0.60	**0.63**	0.62
GEO	0.51	0.51	0.49	0.51	0.49	0.53	**0.54**
UK	0.55	0.58	0.55	0.60	0.55	0.62	**0.64**
Mean rank	2.42	3.08	2.92	4.17	2.58	6.25	**6.58**

The top-performing methods are highlighted for each dataset. The reported C-index is the average C-index of 100 runs of 10-fold cross-validation on each dataset. VIM.SDE and W.SDE are two versions of the SDE method using VIM-time and W-time, respectively. VIM.NET and W.NET are two versions of the NET method using VIM-time and W-time, respectively. OS is overall survival time and RF is relapse-free survival time.

4 Performance for cancer signatures on the validation datasets

We train our model on one dataset and test on the other three independent datasets. We validate whether our signatures are comparable to those signatures in benchmark methods. Considering the sample size and the heterogeneity of datasets, we choose two big and homogeneous datasets TCGA and METABRIC to train. Compared to training on the TCGA dataset, we obtain better results when training on the METABRIC dataset because it has

more training samples than the TCGA dataset. We show the results based on the METABRIC dataset in the main manuscript of this work. We report the results based on the TCGA dataset here because the TCGA dataset is commonly used in breast cancer research. When training on the TCGA dataset, we only use one gene *ASPM* to achieve better results than the current 6 benchmark methods (Table C). Additionally, *ASPM* expression levels have greater prognostic significance than those of *AURKA*, *ESR1*, and *ERBB2* which are used to be independent predictors in breast cancer [11].

Table C: **Performance for cancer signatures on the three validation sets (METABRIC, GEO, and UK).**

	PAM50	Mamma	RS	GGI97	Endo	LM	scP.W	AURKA	ESR1	ERBB2
METABRIC(OS)	0.47	0.54	0.56	0.48	0.56	0.52	**0.57**	**0.57**	0.52	0.47
METABRIC(RF)	0.46	0.56	0.59	0.48	0.58	0.55	0.62	**0.64**	0.58	0.49
GEO	0.50	0.52	**0.58**	0.53	0.55	0.53	0.57	0.56	0.57	0.51
UK	0.50	0.59	0.52	0.57	0.63	0.52	**0.63**	0.50	0.58	0.51
Mean rank	1.25	5.50	7.50	3.88	7.38	4.38	**9.12**	7.00	6.62	2.38

The top-performing result is highlighted for each dataset. The reported C-index is the average C-index of 100 runs of 10-fold cross-validation on each dataset. OS is overall survival time and RF is relapse-free survival time.

Table D: **Performance for cancer signatures on the three validation datasets (TCGA, GEO, and UK).**

	PAM50	Mamma	RS	GGI97	Endo	LM	scP.W
TCGA(RF)	0.51	0.38	0.75	0.61	0.74	0.55	**0.79**
TCGA(OS)	0.50	0.48	**0.65**	0.50	**0.65**	0.58	**0.65**
GEO	0.52	0.50	**0.60**	0.52	0.56	0.52	0.55
UK	0.66	**0.70**	0.68	0.68	0.66	0.55	0.62
Mean rank	2.75	2.50	**6.12**	3.75	5.12	2.75	5.00

The top-performing results are highlighted for each dataset. The reported C-index is the average C-index of 100 runs of 10-fold cross-validation on each dataset. OS is overall survival time and RF is relapse-free survival time.

scP.W has the best results of prediction overall survival and relapse-free time on the TCGA dataset. From Table D, we can see that our signatures still have favorable performance compared to the signatures from the benchmark methods.

The KM curve and the Log-rank test of risk group prediction using scP.W have shown that scP.W successfully stratifies patients in TCGA into two risk groups of relapse and overall survival. Fig A shows the KM curve and the Log-rank test of risk group prediction using other benchmark methods. Regarding relapse risk prediction, RS, Endo, and GGI97 can group patients according to survival difference while PAM50, Mamma, and LM cannot. For overall survival prediction, only RS, Mamma, and Endo result in two risk groups that have significant differences in survival. However, Mamma predicts that the high-risk group patients have high survival probability than low-risk group patients, which is opposite to clinical information.

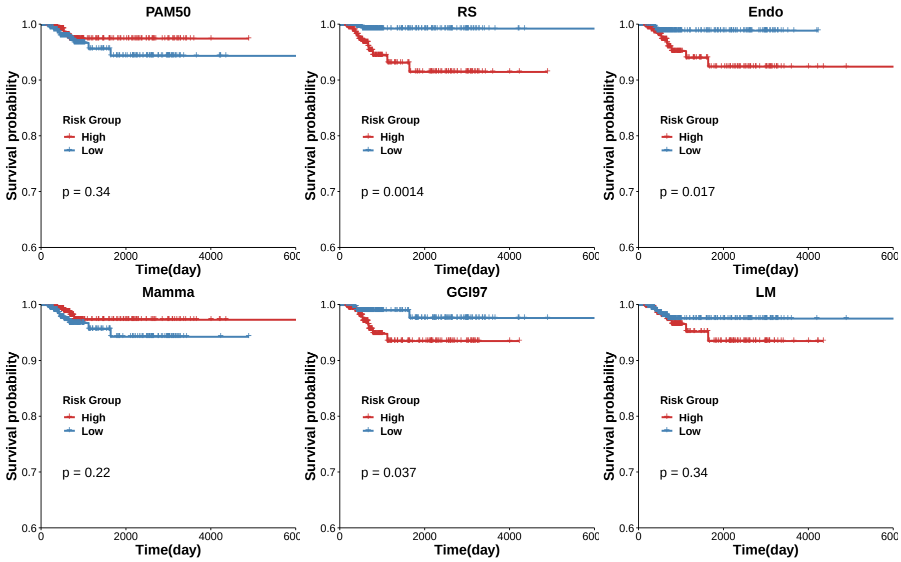

(1)

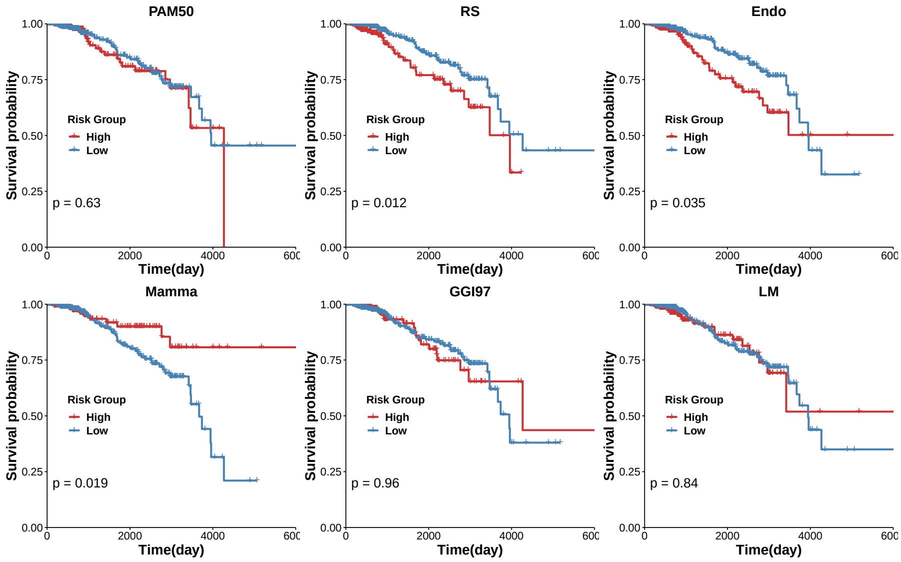

(2)

Figure A: (1)The KM curve and Log-rank test of risk group prediction using benchmark methods on TCGA(RF); (2)The KM curve and Log-rank test of risk group prediction using benchmark methods on TCGA(OS).

5 Optimization of parameters for *scPrognosis*

scPrognosis has four parameters that require tuning: N, the number of selected genes (signatures) used in the Cox PH model, α, β, and γ, the weights

for MAD, SDE, and NET respectively (see Materials and Methods section for a full description of the parameters). Based on the constraint $\alpha+\beta+\gamma=1$, the value of γ depends on the values of α and β. To obtain the breast cancer signatures, we train *scPrognosis* on the METABRIC dataset and perform the grid search method to select the optimized parameters. The optimized parameters are determined by the best average C-index of 100 runs of 10-fold cross-validation. For the ith run of 10-fold cross-validation ($i=1,\dots,100$), we randomly partition the dataset into ten-equal sized sub-datasets. Of the ten sub-datasets, one sub-dataset is retained as the testing data, and the remaining nine sub-datasets are used as training data. Each of the ten sub-datasets takes a turn to be testing data. At the end of the run, ten C-indices are produced, which are averaged to obtain the average C-index for this run of 10-fold cross-validation, denoted as C-index$_i$. We repeat this procedure 100 times and finally we use the average C-index over the 100 runs of 10-fold cross-validation as metric for tuning the parameters of *scPrognosis*.

Specifically, we conduct a grid search by setting the number of selected genes i.e. N from 1 to 60, and the weights α, β, and γ from 0 to 1 with a step of 0.1 and the constraint $\alpha+\beta+\gamma=1$. We aim to obtain the best C-index metric as described above with the lest signatures. Fig B shows the performance of different combinations of parameters. From Fig B1, we can observe that *scPrognosis* has the best C-index when $\alpha=0.4, \beta=0.2$ ($\gamma=0.4$) and when choosing the top 10 gene as breast cancer signatures (Fig B2). The full results of the performed grid search for the optimal parameters can be found online at `https://github.com/XiaomeiLi1/scPrognosis`.

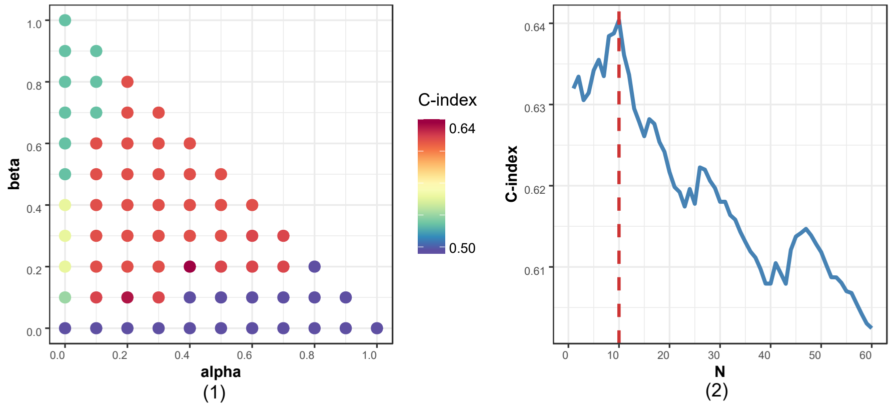

Figure B: **The performance of different combinations of parameters on METABRIC.**(1)The performance of the grid-search for α, and β when $N=10$. The X-axis is α, and the Y-axis is β. The color of a point depends on the value of C-index on its coordinate. (2)The performance of the grid-search for N when $\alpha=0.4$, and $\beta=0.2$. The X-axis is N, and the Y-axis is C-index. The red vertical dash line is $N=10$ where obtains the best C-index.

6 The significant switch-like differential expression of genes along pseudotime

We plot the expression and the maximum likelihood sigmoid fit of two EMT markers and ten cancer signatures to visualize the switch-like behavior of genes during the pseudotime. From the Fig C, we can see that *FXYD3*, *KRT15*, and *KRT6B* switch off at M stage, and *VIM* switch on at M stage. Interestingly, the remain genes are off both the E and M stages but switch on in the hybrid E/M stage, which may give us clues about the link between the hybrid E/M stage and the clinical outcomes of breast cancer.

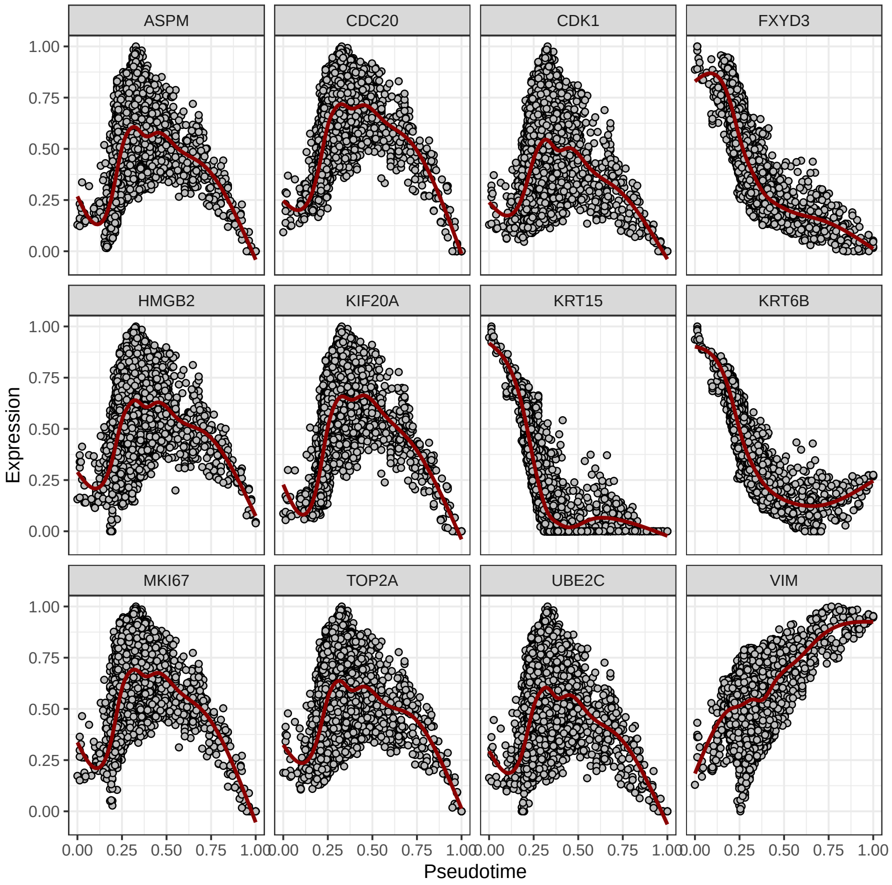

Figure C: **EMT markers and breast cancer signatures expression across the EMT pseudotime.** The X-axis is the EMT pseudotime, and the Y-axis is the gene expression level. A grey point indicates a cell. The red lines are the maximum likelihood sigmoid fit of gene tendencies along the EMT pseudotime.

7 The significant biological process of the inferred signatures

The 10 signatures are significantly enriched in several biological functions. The top 10 functions are known to be critical for cell processes. In addition, ubiquitin-protein ligase activity might be a mechanism to trigger cancer initialize and progress.

Table E: **Gene Ontology mapped biological process for the 10 breast cancer signatures.**

Index	Term	P-value
1	regulation of ubiquitin protein ligase activity (GO:1904666)	6.93E-07
2	positive regulation of ubiquitin protein ligase activity (GO:1904668)	8.10E-06
3	positive regulation of protein ubiquitination involved in ubiquitin-dependent protein catabolic process (GO:2000060)	9.66E-06
4	DNA topological change (GO:0006265)	8.08E-06
5	anaphase-promoting complex-dependent catabolic process (GO:0031145)	7.25E-06
6	negative regulation of ubiquitin protein ligase activity (GO:1904667)	6.71E-06
7	positive regulation of ubiquitin-protein ligase activity involved in regulation of mitotic cell cycle transition (GO:0051437)	6.46E-06
8	regulation of ubiquitin-protein ligase activity involved in mitotic cell cycle (GO:0051439)	5.50E-06
9	negative regulation of ubiquitin-protein ligase activity involved in mitotic cell cycle (GO:0051436)	5.27E-06
10	DNA ligation (GO:0006266)	3.43E-05

The biological process are highly relevant to the cell cycle and ubiquitin protein ligase activity.

References

[1] Parker JS, Mullins M, Cheang MC, Leung S, Voduc D, Vickery T, et al. Supervised risk predictor of breast cancer based on intrinsic subtypes. Journal of clinical oncology. 2009;27(8):1160.

[2] Van't Veer LJ, Dai H, Van De Vijver MJ, He YD, Hart AA, Mao M, et al. Gene expression profiling predicts clinical outcome of breast cancer. nature. 2002;415(6871):530.

[3] Paik S, Shak S, Tang G, Kim C, Baker J, Cronin M, et al. A multigene assay to predict recurrence of tamoxifen-treated, node-negative breast cancer. New England Journal of Medicine. 2004;351(27):2817–2826.

[4] Sotiriou C, Wirapati P, Loi S, Harris A, Fox S, Smeds J, et al. Gene expression profiling in breast cancer: understanding the molecular basis of histologic grade to improve prognosis. Journal of the National Cancer Institute. 2006;98(4):262–272.

[5] Filipits M, Rudas M, Jakesz R, Dubsky P, Fitzal F, Singer CF, et al. A new molecular predictor of distant recurrence in ER-positive, HER2-negative breast cancer adds independent information to conventional clinical risk factors. Clinical Cancer Research. 2011;17(18):6012–6020.

[6] Minn AJ, Gupta GP, Siegel PM, Bos PD, Shu W, Giri DD, et al. Genes that mediate breast cancer metastasis to lung. Nature. 2005;436(7050):518.

[7] Van Dijk D, Sharma R, Nainys J, Yim K, Kathail P, Carr AJ, et al. Recovering gene interactions from single-cell data using data diffusion. Cell. 2018;174(3):716–729.

[8] Bendall SC, Davis KL, Amir EaD, Tadmor MD, Simonds EF, Chen TJ, et al. Single-cell trajectory detection uncovers progression and regulatory coordination in human B cell development. Cell. 2014;157(3):714–725.

[9] Tan TZ, Miow QH, Miki Y, Noda T, Mori S, Huang RYJ, et al. Epithelial-mesenchymal transition spectrum quantification and its efficacy in deciphering survival and drug responses of cancer patients. EMBO molecular medicine. 2014;6(10):1279–1293.

[10] Specht AT, Li J. LEAP: constructing gene co-expression networks for single-cell RNA-sequencing data using pseudotime ordering. Bioinformatics. 2016;33(5):764–766.

[11] Haibe-Kains B, Desmedt C, Sotiriou C, Bontempi G. A comparative study of survival models for breast cancer prognostication based on microarray data: does a single gene beat them all? Bioinformatics. 2008;24(19):2200–2208.
